# Transcatheter Versus Surgical Closure of Atrial Septum Defect: A Debate from a Developing Country

**DOI:** 10.15171/jcvtr.2014.013

**Published:** 2014-12-30

**Authors:** Waleed T Siddiqui, Tariq Usman, Mehnaz Atiq, Muhammad Muneer Amanullah

**Affiliations:** ^1^Dow University of Health Sciences, Karachi, Pakistan; ^2^Department of Cardiothoracic Surgery, The Aga Khan University Hospital (AKUH), Karachi, Pakistan; ^3^Department of Pediatrics, The Aga Khan University Hospital (AKUH), Karachi, Pakistan

**Keywords:** Congenital, ASD, Percutaneous, Surgery

## Abstract

***Introduction:*** This study compares the effectiveness and cost of trans-catheter verses surgical closure of secundum atrial septum defect (ASD). ASD accounts for 10% of congenital cardiac defects. Trans-catheter closure of secundum ASD is increasingly used as the primary intervention. Surgical repair is advised in a proportion of secundum type defects which are unsuitable for device closure.

***Methods:*** We reviewed the clinical course of 176 patients who underwent closure of isolated secundum ASD. The patients were assigned to either the device or surgical group depending upon the treatment they received. Successful closure was assessed immediately after the procedure. The following outcomes were studied: mortality, morbidity, hospital stay, and costs.

***Results:*** Ninety five patients were in the surgical group and 81 patients were in the group undergoing device closure. The median age was 14.0 years (range 1.1-61.0) for surgical group and 24.0 years (range 0.5-68.0) for the device group. The mortality in both groups was 0. The procedure success rate was 100% for the surgical group and 96.3% for the device group. The complication rate was 13.7% for surgical group and 7.4% for the device group. The mean length of hospital stay was 5.0 ± 2.7 days for surgical group and 3.0 ± 0.4 days for device group. The procedure cost for surgery was found to be 12.3% lower than that of trans-catheter closure.

***Conclusion:*** Successful closure is achieved by both methods. Trans-catheter closure results in lower rate of complication and hospital stay but the cost of the procedure tends to be higher than surgery.

## Introduction

Atrial septal defect (ASD) is a common form of congenital heart disease accounting for approximately 10% of all congenital cardiac defects.^1^ Surgical closure has been for many years, the gold standard treatment for patients with ASDs.^2,3^ In 1976 Mills and King reported the first transcatheter closure of secundum ASD^4^, since then transcatheter closure of secundum ASD has evolved over the past few decades and is being increasingly used as the primary intervention in recent years. Surgical repair has been proven to be superior to medical treatment in ostium primum and sinus venosus type ASDs, as well as in a proportion of secundum type defects which are unsuitable for device closure^5^, with a low operative mortality and high long-term survival in middle-aged and elderly patients.^6,7^

The diagnosis and management of ASD in both pediatric and adult populations have benefited greatly from major advances over the last few years^8^ including 3 dimensional and intracardiac echocardiography, as well as percutaneous device closure. Despite these advances, ASDs remain “the most under diagnosed congenital heart disease in the adult age group”, with age at diagnosis being clearly linked to subsequent complications including the late development of pulmonary hypertension and atrial dysrhythmias.^9^

As part of the general evolution of techniques in cardiac surgery, emphasis has been placed on shorter incisions and other surgical methods proposed as “less” or as “minimally” invasive techniques, including minimal access, port access, robotics, video-assisted, etc.^7,10-12^, Complete closure of ASDs has been achieved less frequently with transcatheter techniques than with surgery. On the other hand, surgery results in a significant morbidity.^13,14^ However, there are no single regional and national reports of comparisons of early results, closure rates, mortality, morbidity, and complications between patients treated with either transcatheter or surgical closure of ASD performed at the same time and in the same institution.

The purpose of this study was to compare the safety and efficacy of transcatheter device closure of secundum ASD with concurrent surgical repair results in a single institution.

## Material and methods

### Eligibility Criteria

We reviewed charts of 176 patients who were admitted for closure of secundum ASD either by surgery or device closure between June 2006 to July 2012 and returned for early or late follow-up at the Aga Khan University Hospital. The inclusion criteria for both groups consisted of the presence of an isolated secundum ASD (diameter of ≤33 mm for device group; while no limit for surgery group), a left to right shunt with Q_p_/Q_s_ ratio ≥ 1.5:1 or patients with small defect but history of paradoxical embolism. Excluded from the study were those who had other types of ASD (foramen primum or sinus venosus) or the presence of an additional cardiac malformation [ventricular septal defect (VSD), tetralogy of Fallot, pulmonary stenosis etc.)]amenable only by surgical repair.

Additional exclusion criteria for the percutaneous closure group were patients with multiple defects that were unsuitable by device closure or a defect too close to the superior vena cava, atrioventricular valve, coronary sinus or pulmonary veins (predictable failure of interventional procedure).

Patients who met the inclusion criteria for both groups were given an option for opting either procedure. They were presented with the complications associated with each procedure (intervention with a chance of embolization and surgery with a higher rate of morbidity) before they made their decision.

### Operative Technique

### Surgery

All surgical procedures were done using cardiopulmonary bypass. The chest was opened by via median sternotomy. After total cardiopulmonary bypass had been achieved either cold blood or crystalloid cardioplegic solution was given antegrade through the aortic root. The method used to close the defect depended on its size and anatomical type. Three patients (3.2%) underwent direct suture closure while the rest were closed by autologous pericardial patch with Prolene 5-0 suture.

### Device Implantation

The occlusion device used was the Amplatzer Septal Occluder. This was because it is the only FDA approved available deice in our setting. All percutaneous closure was done under general anesthesia. The right femoral vein was accessed using a 7–8 Fr short sheath. Heparin was administered to achieve an activated clotting time (ACT) > 200 seconds at the time of device deployment along with 1 g cefazolin intravenously. Right heart catheterization was performed to ensure the presence of normal pulmonary vascular resistance. The defect was sized using a 34 mm balloon. The balloon was then inflated with diluted contrast until the left to right shunt ceases as observed by color flow Doppler TEE (flow occlusion). If the defect had adequate rims (>5 mm), we selected a device 0–2 mm larger than the balloon stretched diameter. However, if the superior/anterior rim was deficient (5–7 mm), we selected a device 4 mm larger than the balloon stretched diameter. Our choice for the device size depended on the echocardiographic measurements of the defect (TEE). In adults, a device about 4–6 mm larger than the two-dimensional (2D) size by color Doppler while in children, a device no more than 2 mm larger than the 2D size by color Doppler was used. Device was deployed with guide wire over the left upper pulmonary vein. Position of the device was confirmed on fluoroscopy and TEE. Once the position of the device was confirmed, it was released.

### Outcomes

Patients who had surgical closure residual intra-atrial shunting (significant >2 mm) was examined by colour Doppler while patients who had device closure a transesophageal echocardiogram (TTE) with color Doppler was done immediately after the procedure. In addition a chest radiograph, an ECG and a physical examination was conducted in both groups within 24 h.

### Primary Outcome

If death occurred any time after surgery.

### Secondary Outcome

Presence of any complications such as residual shunt, reintervention, arrhythmias requiring medications, pericardial effusion, etc.

### Statistical Analysis

Data was analyzed using the SPSS for Windows (version 19.0) and is expressed as mean, median and minimum and maximum ranges where appropriate.

### Cost Estimation

Cost per case was calculated based on total hospitalization that was reflected on the bill that patient would have paid, excluding any subvention. This means that it was a true reflection of the actual cost of treatment. The cost included hospital room charges, laboratory investigations, pharmaceutical charges, clinician and anaesthesia charges, facility and treatment charges, cost of surgery (including cardiopulmonary bypass) or device and for their respective length of stay. In doing so, we were able to achieve a meaningful comparison of true costs incurred by each patient for either of the methods, which was the basis of our comparison.

## Results

A total of 176 patients underwent closure of ASD. Eighty-one patients for device closure and 95 patients for surgery satisfied the inclusion criteria and were included as subjects in each arm of study. The median age at operation for the device group was comparatively higher, 24.0 years (range 0.5-68 years) while of the surgery group was 14.0 years (range 1.1 -61 years). A comparison of demographic and preoperative variables is illustrated in Table 1.

**Table 1:** Comparison of demographic and baseline clinical data between surgical and device closure group

	**Mean ± SD**	
	**Surgical Patients (n=95)**	**Device Patients (n=81)**
Height (cm)	130.9 ± 37.3	141.8 ± 26.5
Weight (kg)	40.0 ± 29.1	44.0 ± 21.6
Age (years)	18.8 ± 16.7	23.3 ± 16.4
Patients with family history of ASD	14	3
Pulmonary Hypertension (on admission)	14	7
Size of ASD (mm)	24.7 ± 9.9	18.9 ± 7.0

ASD: atrial septum defect

### Comparison of Closure Results

The device group consisted of 81 patients, 32 males (39.5%) and 49 females (60.5%).Of the 81 patients, 78 had devices successfully deployed (96.3%) across the atrial septum. In 3 (3.7%) of the patients the device embolized soon after implantation and had to be removed surgically. These patients will be discussed subsequently. These 3 patients have been included in both transcatheter and surgical category since they had an immediate successful closure but due to embolization were later surgically treated. There were 11 candidates who had been initially considered suitable for device closure based on transthoracic echocardiography. However, device implantation was not attempted in these subjects since on trans-esophageal echocardiography the ASD was found to have a deficient rim (one of either inferior, superior or aortic rim) and were thus deferred for surgery (the outcome of these patients is included under the surgical data only). The mean size of the ASD was 18.9 ± 7.0 mm (range 4 to 33 mm). The mean size of the amplatzer device was 23.4 ± 6.7 mm (range 8 to 34 mm).The mean fluoroscopy time was 14.5 ± 10.3 min (range 3.2 to 54 min).

The surgical group consisted of 95 patients, 50 males (52.6%) and 45 females (47.4%). The size of the defect ranged from 7 mm to 63 mm, mean 25.2 ± 10.0 mm. The mean cardiopulmonary bypass time was 58.7 ± 23.4 min and the aortic cross-clamp time was 32.1 ± 15.3 min. The mean minimum temperature reached was found to be 35.5 ^o^C. After each surgery, a postoperative on table echo was done to check for the presence of any hemodynamic abnormality or for any residual patch defect (significant >2 mm). After the procedure inotropic support was provided in 50 (52.6%) cases and consisted of either one of epinephrine, dopamine, milrinone, dobutamine or a combination of epinephrine and milrinone.

All but one patient in the surgical group had a successful closure immediately after the procedure.

A comparison of operative and postoperative outcomes between the two groups is demonstrated in Table 2.

**Table 2:** Comparison of operative and postoperative outcomes between surgical and device closure group

	**Mean ± SD**	
	**Surgical Patients (n=95)**	**Device Patients (n=81)**
Duration of Surgery/Procedure (min)	168.3 ± 38.1	64.2 ± 29.0
CPB Time/Fluoroscopy Time (min)	58.7 ± 23.4	14.5 ± 10.3
Aortic Cross-Clamp Time (min)	32.1 ± 15.3	-
Mean Minimum Temperature (^o^C)	35.5 ± 1.8	-
Length of CICU stay (days)	1.5 ± 1.1	-
Length of hospital stay (days)	5.0 ± 2.7	3.0 ± 0.4
Residual ASDs (≥2 mm)	0	0
On table deferred for surgery	0	11
Total procedure success (%)	100	96.3
Mean total cost (USD $)	2460.90 ± 443.57	2764.61 ± 528.07

ASD: atrial septum defect; CPB: cardiopulmonary bypass time

### Comparison of ICU, Hospital Stay and Procedure Cost

In the device group all patients were shifted immediately after percutaneous closure from the cardiac catheterization laboratory to the wards. In contrast all surgical patients were required to be in the intensive care unit for at least one day.

The mean length of inpatient stay for the surgical group was 5.0 ± 2.7 days. This was longer than the patients of device group 3.0 ± 0.4 days.

The mean cost per successful closure procedure was around USD $300 higher for the device. Although the cost of radiology and laboratory investigations was higher in the surgical group the high cost of the Amplatzer device (currently USD $2500) weighted much heavier on the overall total charges despite patients in this group had a shorter hospital stay.

### Comparison of Complications

There were complications observed in both surgical and device closure patients. Table 3 summarizes the major complications encountered between the two groups.

**Table 3:** Comparison of major complications between surgical and device closure group

	**Surgical Patients (n=95)**	**Device Patients (n=81)**
Morbidity		
Complications (%) [on patient-basis]	13.7	7.4
Wound Infections/Groin Hematoma	4	0
Pneumonia	1	0
Pericardial Effusion (requiring tube thoracostomy)	0	0
Pleural Effusion (requiring tube thoracostomy)	2	0
Pneumothorax (requiring tube thoracostomy)	2	0
Reopening	1	0
Device Embolization	0	3
Device Endocarditis	-	0
Arrhythmias (requiring medication)	4	3
Readmission within 30 days)	3	1

In the surgery group there were two patients of each pleural effusion and pneumothorax. These were treated via mechanical drainage and developed no further complication. There was an immediate early surgical wound complication in one patient requiring sternal wire removal and antibiotic treatment. There was one patient which required a reopening, due to misdirection of the IVC to the left atrium resulting in post-operative desaturation. The patient was taken back to surgery and the ASD was closed with IVC draining into the right atrium. There were 3 readmissions within 30 days which were due to superficial wound infection. The mean time for follow-up in the surgical group was 6.8±9.6 months. Of the 95 patients only 8 (8.4%) were in NYHA II on follow-up and the rest were in NYHA I.

In comparison, patients in device group had a much lower rate of complication. Device embolization requiring surgical retrieval was the most major complication and occurred in 3 patients, shortly after the procedure. Cardiac arrhythmia was a common minor complication of both groups occurring in 3 patients in device group and 4 patients in the surgery group. These were brief episodes and were resolved by the use of medication. There were no cases of vascular complication (groin hematoma) in patients treated by cardiac intervention. There was one patient who was readmitted due to non-cardiac reasons.

There were no deaths in either the device or surgical group and all patients were discharged in stable conditions from the hospital.

Of the 3 devices that were dislodged, one of them was a 16 year old girl who developed premature ventricular contractions shortly after the procedure. Echocardiogram and fluoroscopy revealed the device to be in the right ventricle near the tricuspid valve requiring immediate surgical retrieval. The second was a 53-year-old female. Her pre-discharge echo showed an ASD and fluoroscopy revealed the device in the left ventricle. The last case was a 16-year-old boy who experienced post-operative palpitations. Echocardiogram revealed the device to be dislodged in right ventricular outflow tract. All these patients were immediately operated surgically. The defect was closed using an autologous pericardial patch and postoperative echo showed no residual ASD. All of them made a smooth postoperative recovery and were discharged in stable conditions. On follow-up (2 years, 2 years and 3.5 months) they were asymptomatic and there was no residual ASD demonstrated by echocardiography.

The mean time for follow-up for this group was 10.8 ± 15.9 months. Out of the 81 patients only 5 (6.2%) were in NYHA II while the rest were in NYHA I.

## Discussion

ASDs account for 10% of all cardiac malformations in childhood.^1^ If left uncorrected, they may lead to premature death from congestive cardiac failure.^15^ Patients with ASD and left to right shunts are at increased risk of developing pulmonary arterial hypertension.^16^ As seen in our data 21 patients (14 in surgical and 7 in device patients), both of pediatric and adult age group had developed pulmonary hypertension at the time of presentation. These were echocardiographic findings and the outcome of these patients improved after treatment.

ASD has been surgically repaired for almost 60 years.^17^ However in recent years the transcatheter approach to occlude secundum ASD has gained considerable popularity. The main advantages of device include: the self-centering mechanism, leading to better complete closure rates; delivery through relatively small introducing sheaths; and simple placement technique and retrievability before release. In addition, there are fewer complications, avoidance of sternotomy and cardiopulmonary bypass, shorter hospital stay, reduced need for blood products and less patient discomfort.

This retrospective study describes the advantages and complications of each technique. Regardless of the method the mortality in both groups was found to be zero. However, one of the main finding of our study was that the morbidity was higher in surgical group compared to the device group. This finding is in accordance with other studies.^18,19^ Although age was not a considered a criterion for selection for either procedure, patients in the surgical group were relatively much younger and had comparatively larger size of defects which could have attributed to the complications.

Despite the fact that device closure tends to have fewer complications, embolization is a potentially feared one which requires immediate surgical intervention for retrieval and correction of defect.^20^ In our institution this complication cannot be attributed by our learning curve since it occurred late in the course, after implantation of more than 50 devices.

The 11 patients that were initially assessed to be suitable for device closure on transthoracic echocardiography but in the cardiac catheterization lab, on transesophageal echocardiography they were found to have a deficient rim. One may question as why we did not do a TEE earlier. The reason is being that a TEE is much more expensive than TTE and requires the patient to undergo general anesthesia. This is a significant financial concern in a third world country like Pakistan and hence in our initial evaluation, TTE is used as the primary diagnostic tool.

In our study, on follow-up in the device group there were no patients who developed device endocarditis which has been described as a late complication of this procedure.^21^

Our analysis revealed that mean surgical closure of ASD was USD $ 2460.90 ± 443.57 while of the device group was $ 2764.61 ± 528.07 (these costs are inclusive of all expenses: cost of device/cardiopulmonary bypass, laboratory, pharmaceutical and radiology charges, operating room costs etc.) revealing surgery to be 12.3% cheaper. This is in correlation with another study by Quek et al^24^ where the mean cost per successful procedure was considerably higher for the device group. The difference is largely due to the high cost of the device. Other devices might be cheaper and may lead to a lower cost of the transcatheter procedure but only the Amplatzer was used in our setting.

Nevertheless, there is less utilization of beds and ICU facilities which reduces load on hospital resources and ancillary staff.

The monetary difference is significantly important in a low-income country like Pakistan where health care resources are limited and funds must be allocated in a way which allows more number of patients to be effectively treated.

### Clinical Implication

In our study device closure of ASD seems comparable to surgery when the anatomy of the ASD is suitable. Device closure technique requires a shorter hospital stay implying early resumption of school or work. Surgical closure is of course possible for all sizes and anatomical variants of ASDs. Open heart surgery has its inherent risk associated but the incidence of major complications (device failure and embolization) are the same in the two groups.

## Limitations

The study had some limitations. When both techniques were available, patients treated surgically were those with multiple or more complex defects. This could impact on the occurrence of complications.

The sample size of the study population was small and this was a single-center study, so the results are limited to our own experience.

## Conclusion

We conclude that in experienced hands, transcatheter ASD closure provides comparable results to surgery though with a shorter hospital stay. However, device closure of ASD should only be done in centers where an immediate surgical backup is available. This is the first study of its kind evaluating outcomes and costs from Pakistan.

## Ethical issues

The study was approval by the Local Ethics Committee.

## Competing interests

Authors declare no conflict of interests in this study.
